# Supplementary material for: Unveiling the Bioactive Potential of the Invasive Jellyfish Phyllorhiza punctata Through Integrative Transcriptomic and Proteomic Analyses
Source: Biomolecules. 2025 Aug 4;15(8):1121. doi: 10.3390/biom15081121 (PMC12383608; doi:10.3390/biom15081121)
Supplement: Supplementary file 1 [file biomolecules-15-01121-s001.zip › Suplementary Material/FileS3/PEPFOLD4/XP_065067169_8734/clusters.html]

 PEP-FOLD clusters


Download archive of all models  here   

|  |  |  |
| --- | --- | --- |
| # | file | sOPEP |
| # cluster 1 - Size: 88 |  |  |
|  | XP\_065067169\_8734-model1 | -15.5529 |
|  | XP\_065067169\_8734-model1.1 | -15.5529 |
|  | XP\_065067169\_8734-model1.2 | -15.5439 |
|  | XP\_065067169\_8734-model1.3 | -15.5439 |
|  | XP\_065067169\_8734-model1.4 | -15.5439 |
|  | XP\_065067169\_8734-model1.5 | -15.5439 |
|  | XP\_065067169\_8734-model1.6 | -15.5439 |
|  | XP\_065067169\_8734-model1.7 | -15.5439 |
|  | XP\_065067169\_8734-model1.8 | -15.5439 |
|  | XP\_065067169\_8734-model1.9 | -15.5439 |
|  | XP\_065067169\_8734-model1.10 | -15.5439 |
|  | XP\_065067169\_8734-model1.11 | -15.5439 |
|  | XP\_065067169\_8734-model1.12 | -15.5439 |
|  | XP\_065067169\_8734-model1.13 | -15.5346 |
|  | XP\_065067169\_8734-model1.14 | -15.5346 |
|  | XP\_065067169\_8734-model1.15 | -15.5346 |
|  | XP\_065067169\_8734-model1.16 | -15.5346 |
|  | XP\_065067169\_8734-model1.17 | -15.5346 |
|  | XP\_065067169\_8734-model1.18 | -15.5346 |
|  | XP\_065067169\_8734-model1.19 | -15.5346 |
|  | XP\_065067169\_8734-model1.20 | -15.5346 |
|  | XP\_065067169\_8734-model1.21 | -15.5346 |
|  | XP\_065067169\_8734-model1.22 | -15.5346 |
|  | XP\_065067169\_8734-model1.23 | -15.5346 |
|  | XP\_065067169\_8734-model1.24 | -15.5088 |
|  | XP\_065067169\_8734-model1.25 | -15.5088 |
|  | XP\_065067169\_8734-model1.26 | -15.5088 |
|  | XP\_065067169\_8734-model1.27 | -15.5088 |
|  | XP\_065067169\_8734-model1.28 | -15.5088 |
|  | XP\_065067169\_8734-model1.29 | -15.5088 |
|  | XP\_065067169\_8734-model1.30 | -15.5088 |
|  | XP\_065067169\_8734-model1.31 | -15.4961 |
|  | XP\_065067169\_8734-model1.32 | -15.4961 |
|  | XP\_065067169\_8734-model1.33 | -15.4961 |
|  | XP\_065067169\_8734-model1.34 | -15.4961 |
|  | XP\_065067169\_8734-model1.35 | -15.4961 |
|  | XP\_065067169\_8734-model1.36 | -15.4842 |
|  | XP\_065067169\_8734-model1.37 | -15.4842 |
|  | XP\_065067169\_8734-model1.38 | -15.4842 |
|  | XP\_065067169\_8734-model1.39 | -15.4842 |
|  | XP\_065067169\_8734-model1.40 | -15.4842 |
|  | XP\_065067169\_8734-model1.41 | -15.4842 |
|  | XP\_065067169\_8734-model1.42 | -15.4842 |
|  | XP\_065067169\_8734-model1.43 | -15.4842 |
|  | XP\_065067169\_8734-model1.44 | -15.4842 |
|  | XP\_065067169\_8734-model1.45 | -15.4842 |
|  | XP\_065067169\_8734-model1.46 | -15.4842 |
|  | XP\_065067169\_8734-model1.47 | -15.4822 |
|  | XP\_065067169\_8734-model1.48 | -15.4822 |
|  | XP\_065067169\_8734-model1.49 | -15.4822 |
|  | XP\_065067169\_8734-model1.50 | -15.4822 |
|  | XP\_065067169\_8734-model1.51 | -15.4432 |
|  | XP\_065067169\_8734-model1.52 | -15.4432 |
|  | XP\_065067169\_8734-model1.53 | -15.4432 |
|  | XP\_065067169\_8734-model1.54 | -15.4416 |
|  | XP\_065067169\_8734-model1.55 | -15.4416 |
|  | XP\_065067169\_8734-model1.56 | -15.4416 |
|  | XP\_065067169\_8734-model1.57 | -15.4416 |
|  | XP\_065067169\_8734-model1.58 | -15.4416 |
|  | XP\_065067169\_8734-model1.59 | -15.4416 |
|  | XP\_065067169\_8734-model1.60 | -15.4416 |
|  | XP\_065067169\_8734-model1.61 | -15.4416 |
|  | XP\_065067169\_8734-model1.62 | -15.4416 |
|  | XP\_065067169\_8734-model1.63 | -15.4416 |
|  | XP\_065067169\_8734-model1.64 | -15.4416 |
|  | XP\_065067169\_8734-model1.65 | -15.4416 |
|  | XP\_065067169\_8734-model1.66 | -15.4416 |
|  | XP\_065067169\_8734-model1.67 | -15.4416 |
|  | XP\_065067169\_8734-model1.68 | -15.4416 |
|  | XP\_065067169\_8734-model1.69 | -15.4416 |
|  | XP\_065067169\_8734-model1.70 | -15.4416 |
|  | XP\_065067169\_8734-model1.71 | -15.4416 |
|  | XP\_065067169\_8734-model1.72 | -15.4416 |
|  | XP\_065067169\_8734-model1.73 | -15.4324 |
|  | XP\_065067169\_8734-model1.74 | -15.4324 |
|  | XP\_065067169\_8734-model1.75 | -15.4324 |
|  | XP\_065067169\_8734-model1.76 | -15.4308 |
|  | XP\_065067169\_8734-model1.77 | -15.4182 |
|  | XP\_065067169\_8734-model1.78 | -15.4182 |
|  | XP\_065067169\_8734-model1.79 | -15.399 |
|  | XP\_065067169\_8734-model1.80 | -15.399 |
|  | XP\_065067169\_8734-model1.81 | -15.399 |
|  | XP\_065067169\_8734-model1.82 | -15.399 |
|  | XP\_065067169\_8734-model1.83 | -15.399 |
|  | XP\_065067169\_8734-model1.84 | -15.399 |
|  | XP\_065067169\_8734-model1.85 | -15.3953 |
|  | XP\_065067169\_8734-model1.86 | -15.3883 |
|  | XP\_065067169\_8734-model1.87 | -15.3883 |
| # cluster 2 - Size: 9 |  |  |
|  | XP\_065067169\_8734-model2 | -15.5372 |
|  | XP\_065067169\_8734-model2.1 | -15.5372 |
|  | XP\_065067169\_8734-model2.2 | -15.5372 |
|  | XP\_065067169\_8734-model2.3 | -15.5372 |
|  | XP\_065067169\_8734-model2.4 | -15.4334 |
|  | XP\_065067169\_8734-model2.5 | -15.4245 |
|  | XP\_065067169\_8734-model2.6 | -15.4245 |
|  | XP\_065067169\_8734-model2.7 | -15.4027 |
|  | XP\_065067169\_8734-model2.8 | -15.4027 |
| # cluster 3 - Size: 3 |  |  |
|  | XP\_065067169\_8734-model3 | -15.4607 |
|  | XP\_065067169\_8734-model3.1 | -15.4607 |
|  | XP\_065067169\_8734-model3.2 | -15.4287 |
|  |  |  |
